# Liver Transcriptome Shows Differences between Acute Hypoxia-Tolerant and Intolerant Individuals of Greater Amberjack (*Seriola dumerili*)

**DOI:** 10.3390/ani13172717

**Published:** 2023-08-26

**Authors:** Duo Li, Yang Yang, Tong Wang, Weiwei Zhang, Sijie Hua, Qingxin Ruan, Xi Wang, Chunhua Zhu, Zining Meng

**Affiliations:** 1 State Key Laboratory of Biocontrol, Institute of Aquatic Economic Animals and Guangdong Province Key Laboratory for Aquatic Economic Animals, School of Life Sciences, Sun Yat-Sen University, Guangzhou 510275, China; liduo7@mail2.sysu.edu.cn (D.L.); yangy888@mail.sysu.edu.cn (Y.Y.); sysuwangtong@163.com (T.W.); zhangww79@mail2.sysu.edu.cn (W.Z.); huasj@mail2.sysu.edu.cn (S.H.); ruanqx3@mail2.sysu.edu.cn (Q.R.); 2 Area of Ecology and Biodiversity, School of Biological Sciences, University of Hong Kong, Hong Kong SAR 999077, China; u3009279@connect.hku.hk; 3 Guangdong Research Center on Reproductive Control and Breeding Technology of Indigenous Valuable Fish, Species, Fisheries College, Guangdong Ocean University, Zhanjiang 524088, China; chz416@163.com; 4 Southern Marine Science and Engineering Guangdong Laboratory, Zhanjiang 524025, China

**Keywords:** greater amberjack (*Seriola dumerili*), acute hypoxia, transcriptome analysis, liver tissue

## Abstract

**Simple Summary:**

The greater amberjack (*Seriola dumerili*) is an economically important fish with high farming value. This species is intolerant to hypoxia, which makes it susceptible to mass mortality and hinders the progress of amberjack cultivation. Based on a comparative analysis of the liver transcriptome between acute hypoxia-tolerant (HT) and intolerant (HS) groups, this study first explored the molecular mechanisms of acute hypoxia in greater amberjack. The results showed that the glycolipid metabolism, antioxidant activity, and apoptotic pathways of greater amberjack significantly responded when exposed to acute hypoxia. In addition, the relative downregulation of apoptosis and autophagy-related genes, such as *endog*, *hm13*, and *casp6*, was also detected in the HT group. The NF-kB pathway was partly promoted in the HT group relative to the HS group to resist apoptosis. This investigation will offer significant technical assistance in the prevention of death caused by acute hypoxia and the subsequent reduction in financial losses.

**Abstract:**

Acute hypoxia is a common abiotic stress in commercial aquaculture and has significant effects on fish physiology and metabolism. Due to its large size and rapid growth, the greater amberjack (*Seriola dumerili*) is an economically important fish with high farming value. This species is intolerant to hypoxia, which makes it susceptible to mass mortality and hinders the progress of amberjack cultivation. Based on a comparative analysis of the liver transcriptome between acute hypoxia-tolerant (HT) and -intolerant (HS) groups, this study first explored the molecular mechanisms of acute hypoxia in greater amberjack. By simulating the acute hypoxic environment and using RNA sequencing (RNA-Seq), the differences in liver transcriptional changes between the acute hypoxia-tolerant (HT) and hypoxia-intolerant (HS) groups of greater amberjack were probed. Based on differential expression analysis, 829 differentially expressed genes (DEGs) were screened in both groups. Relative to the HS group, 374 DEGs were upregulated and 455 were downregulated in the HT group. Compared with the HS group, genes such as *slc2a5* and *prkaa2* related to promoting sugar transport and inhibiting lipid syntheses were upregulated, while genes that inhibit gluconeogenesis and promote lipid syntheses, such as *pgp* and *aacs*, were downregulated. The expression of *odc1* was significantly and relatively downregulated in the HT group, which would lead to the inhibition of intracellular antioxidant activity and decreased scavenging of ROS. The NF-kB pathway was also promoted to some extent in individuals in the HT group relative to the HS group to resist apoptosis. In addition, the relative downregulation of apoptosis and autophagy-related genes, such as *endog*, *hm13*, and *casp6*, was also detected in the HT group. The present findings first reported the regulation mechanism by which liver tissue coped with the acute hypoxia stress in greater amberjack, which will provide important technical support for preventing acute hypoxia-induced death in advance and reducing economic losses.

## 1. Introduction

Oxygen is necessary to maintain the normal survival of almost all organisms. Aquatic organisms are more vulnerable to hypoxic environments than terrestrial organisms [1]. Due to the low solubility of oxygen in water, small changes in the level of dissolved oxygen content may have a significant impact on animals living in oxygen-limited environments [2]. Dissolved oxygen levels in aquatic environments are often influenced by various factors such as eutrophication, global warming, wind speed, diurnal rhythms, and seasonal changes [3]. In recent years, the expansion of aquaculture range and the increase in stocking density accompanied by high levels of nutrient input have resulted in the frequent occurrence of hypoxia in aquaculture environments [4]. Dissolved oxygen levels are one of the most critical aquatic environmental factors; they can adversely affect the growth, reproduction, behavioral activities, and survival of fish [5]. Chronic hypoxia often leads to a decrease in the growth rate of fish, while acute hypoxia poses a great threat to the survival of aquatic organisms and causes huge economic losses [6]. Hypoxia often comes rapidly and unpredictably, eventually leading to mass mortality and seriously affecting farming benefits. Research on the molecular mechanism of acute hypoxia in fish is necessary to address mass mortality and enhance farming efficiency.

In previous studies, several physiological and metabolic changes were found to occur in fish under acute hypoxic conditions, including a decrease in protein synthesis [7], promotion of anaerobic respiration, and sugar xenobiotic pathways [8]. Large amounts of reactive oxygen species (ROS) are also produced when the stress from hypoxia prevents cells from maintaining normal function, leading to apoptosis [9,10]. To date, the molecular mechanisms associated with adaptation to acute hypoxia have been studied in many fish species. For example, acute hypoxia tolerance studies were conducted in rainbow trout (*Oncorhynchus mykiss*) [11], largemouth bass (*Micropterus salmoides*) [12], and yellow catfish (*Pelteobagrus fulvidraco*) [13] and discovered the impact on metabolism and the alteration of antioxidant capacity in fish.

Greater amberjack (*Seriola dumerili*), which lives in the pelagic zone of temperate and subtropical oceans, is naturally distributed mainly in the Australian region, southern Japan, and the East China Sea. Individual growth is rapid, even superior to that of Atlantic salmon (*Salmo salar*) [14]. With firm flesh, uniform fat content, and a sweet taste, it is one of the top three ingredients for high-class sushi restaurants in Japan. The late sexual maturity reduces the nutrient consumption of greater amberjack due to gonadal development to a certain extent, and it can be marketed before the growth is negatively affected by this, thus avoiding the reduction in farming benefits [15]. Therefore, greater amberjack has great advantages and prospects for development in aquaculture. Juveniles of greater amberjack have a very pronounced aggregation habit [16], adults swim fast and consume a lot of oxygen, and individuals at all stages are vulnerable to acute hypoxia. This is very detrimental to the aquaculture industry and not only affects the growth and reproduction of greater amberjack but also causes difficulties and limits the development of large-scale aquaculture. Previously, research on greater amberjack has focused on breeding techniques [14], culture methods [17], feed nutrition [18], and disease prevention and control [19], but no research has been conducted on hypoxic stress. This has led to a lack of understanding about the response of greater amberjack to acute hypoxia conditions, and effective strategies to deal with such issues have not been found.

In recent years, the continuous development of transcriptome sequencing technology (RNA-Seq) has led to an increasingly sophisticated approach to exploring the molecular mechanisms of fish responses to hypoxic environments at the genetic level [20]. For example, Liang et al. (2022) found that pearl gentian grouper (a hybrid of *Epinephelus lanceolatus* ♂ and *Epinephelus fuscoguttatus* ♀) improved its glucose uptake during acute hypoxia by increasing the amount of glucose transporter proteins, thereby promoting glycolysis. Genes involved in fatty acid synthesis were also found to be downregulated during hypoxic exposure, and the upregulation of apoptosis-related genes was detected [21]. In contrast, a study by Tian et al. (2020) pointed out the important role of inhibition of translation and protein synthesis processes in fish adaptation to hypoxic environments [22]. Studies have also reported the effects of acute hypoxia on muscle function and immune-related enzyme activities in Siberian sturgeon (*Acipenser baerii*), as well as the regulation of antioxidant capacity and anaerobic metabolism in response to hypoxic stress [23]. Cumulatively, transcriptomic investigations have illuminated alterations in vital biological mechanisms within fish when subjected to hypoxic conditions. These encompass modifications in energy metabolism, oxidative defense, cell cycle regulation, growth and development, as well as signal transduction.

In this study, we simulated the unpredictable and frequently occurring acute hypoxic conditions in culture, sequenced the greater amberjack liver transcriptome using Illumina sequencing technology, and explored its differential tolerance to acute hypoxia. Differentially expressed genes (DEGs) between tolerant and intolerant groups under acute hypoxic exposure were screened to explore the key pathways regulating hypoxia and provided a basis for the molecular mechanisms that generate differences in tolerance levels under acute hypoxic conditions in greater amberjack.

## 2. Materials and Methods

### 2.1. Ethics Statement

All experiments were performed according to the Guidelines for the Care and Use of Laboratory Animals in China. All experimental procedures and sample collection in the present study were approved by the Animal Care and Use Committee at School of Life Sciences, Sun Yat-Sen University (protocol code SYSU-IACUS-2022-B0129).

### 2.2. Fish Sampling and Hypoxia Treatment

A total of 300 greater amberjack samples were used for anoxia exposure experiments, with a random mix of males and females, and the average body length and weight were 9.62 ± 1.09 cm and 18.03 ± 5.99 g (Table S1). The samples are randomly selected descendants of hybrid breeding populations. They were temporarily reared in net cages and had a 24 h fast before the experiment to ensure the stability of their physiological condition. All of the samples were obtained from Dongshan Island, Fujian, China.

The greater amberjack has high oxygen consumption. To simulate an environment of acute hypoxia, all samples were placed in a 75 L water tank with oxygen supplementation stopped. Under such circumstances, it is currently experiencing a state of acute hypoxia. Therefore, the method of natural depletion of oxygen was used to lower the dissolved oxygen level. Within 15 min, the oxygen level decreased to 1.8 ± 0.2 mg/L, while the greater amberjack lost equilibrium in the body. This indicates that the acute hypoxia tolerance limit has been reached. This finding is consistent with previous research on the fellow member of greater amberjack in *Carangidae* [24]. The first six individuals out of equilibrium were considered to be the hypoxia-sensitive (HS) group, and the last six were considered to be the hypoxia-tolerant (HT) group. The livers of each individual in both groups were sampled, and a total of 12 samples were immediately placed in an RNA Keeper Tissue Stabilizer (Vazyme, Guangzhou, China) for preservation and stored at −80 °C until processing.

### 2.3. RNA Sequencing

Total RNA was extracted from each liver tissue using a TRIzol Reagent Kit (Invitrogen, Carlsbad, CA, USA) according to the manufacturer’s protocol. RNA integrity was assessed using an RNA Nano 6000 Assay Kit of the Bioanalyzer 2100 system (Agilent Technologies, Santa Clara, CA, USA). After quality testing, certified samples from 12 liver tissues were used for library construction and Illumina RNA-Seq, and library quality was assessed on the Agilent Bioanalyzer 2100 system.

The library preparations were sequenced on an Illumina Novaseq platform, and raw data (raw reads) in fastq format were generated. Then, the raw data, which contained adapters and poly-N, were removed, and low-quality reads from the raw data were also eliminated with FastQC. At the same time, the clean data’s Q20, Q30, and GC content were calculated.

### 2.4. Differential Expression Analysis and Enrichment Analysis

The clean data were mapped utilizing the assembled greater amberjack genome (NCBI Reference Sequence: NC_016870.1) [25] as a reference. Hisat2 v2.0.5 [26] was used to build an index of the reference genome and align the paired-end clean reads to the reference genome. To obtain the expression level of each transcript, FeatureCounts v1.5.0-p3 [27] was used to count the reads numbers to map each gene. Then, the fragments per kilobase of transcript per million fragments mapped (FPKM) of each gene were calculated based on the length of the gene and the reads count mapped to the gene. Differential gene expression analysis of two groups (HS and HT) was performed using the DESeq2 R package (1.20.0) [28]. The results with *p*-value < 0.05 and |log_2_ (Fold Change)| > 1 were identified as differentially expressed genes (DEGs). Gene Ontology (GO) and KEGG terms of DEGS were used to further identify relevant biological functions and biological pathways. Significantly enriched GO terms (*p* < 0.05) and KEGG pathways (*p* < 0.05) were determined by ultrageometric test.

### 2.5. Real-Time Quantitative PCR (qRT-RCR) Validation

To verify the accuracy of the sequencing results, 9 DEGs were randomly selected for qRT-PCR in two groups. The given primers were designed by Primer 3 based on the transcripts obtained by sequencing (Table S2). The total RNA of liver samples from all groups was extracted using a TRIzol Reagent Kit, and cDNA was obtained by reverse transcription according to the instructions provided with an RT SuperMix for qPCR (+gDNA wiper) kit (Vazyme, Guangzhou, China). The 10 μL qRT-PCR system included 5 μL of ChamQ SYBR qPCR Master Mix (GDSBIO, Guangzhou, China), 0.2 μL of each primer (10 μM), 1 μL of template cDNA, and 3.6 μL of ddH_2_O. LightCyeler 480 Multiwell Plate 384 (Roche, Ludwigshafen, Germany) was applied for qRT-PCR, and the reaction program was as follows: pre-denaturation at 95 °C for 30 s, followed by 40 cycles of 95 °C for 3 s and 60 °C for 30 s. Three reactions per sample were performed, and *gapdh* was used as an internal reference gene to normalize the relative expression levels of all verified genes. Relative expression levels of genes were calculated using the 2^−ΔΔCt^ method, and plots were generated using Origin 2019b (OriginLab Northampton, Northampton, MA, USA).

## 3. Results

### 3.1. Sequencing Quality and Annotation Results

The 12 liver samples of greater amberjack were used for RNA-seq, and 1,694,368,132 raw reads were generated. The number of clean reads in each library ranged from 41,775,200 to 49,200,360. The cleaned reads were compared with the reference genome. The sequencing statistics of the HS and HT groups were Q20: 95.8–96.9%; Q30: 90.22–91.87%; GC content: 45.54–52.1%; and error rate: 0.030% (Table 1). Cleaned reads from each library were aligned against the reference genome to obtain mapped reads, with about 93.28–94.88% of unigenes in each sample successfully mapping to the reference genome. The sequencing statistics and mapping results for the 12 libraries are shown in Table 1. These results showed that the sequencing data obtained were reliable for use in further analyses. Raw sequence read data were submitted to the NCBI Sequence Read Archive (SRA) under accession number PRJNA990831.

### 3.2. Differentially Expressed Gene Analysis

**Figure 1:**
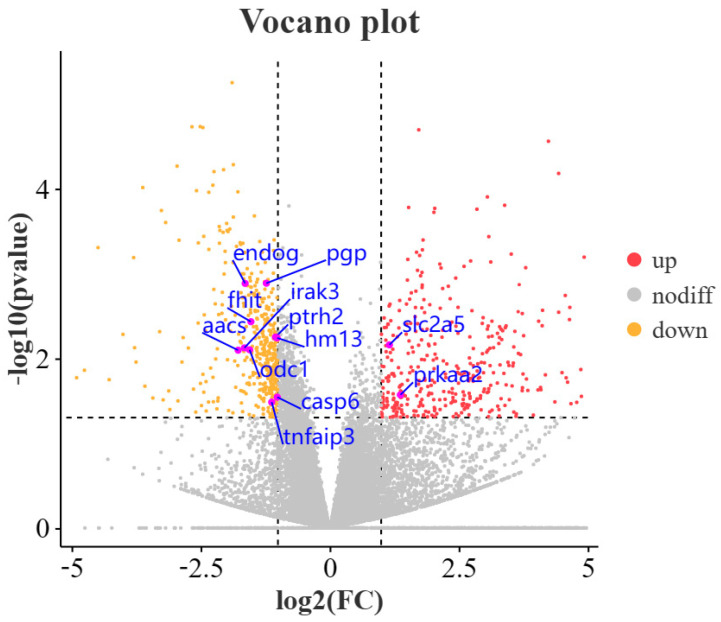
Gene expression profiles in the liver. Differentially expressed genes (DEGs) were shown in red and yellow. Genes that did not exhibit changes in expression were shown in gray.

To identify differentially expressed genes (DEGs) in the HS and HT groups, all 20,796 predicted genes were used to calculate the gene expression levels via the FPKM method (Table S3). We used *p*-value < 0.05 and |log_2_ (Fold Change)| > 1 as criteria to filter genes with significant differential expression between the HS and HT groups. In the HS and HT groups, 829 DEGs were identified. There were 374 upregulated genes and 455 downregulated genes (Figure 1 and Table S4).

### 3.3. Overview of Differential Expression Genes and Enrichment Analysis

**Figure 2:**
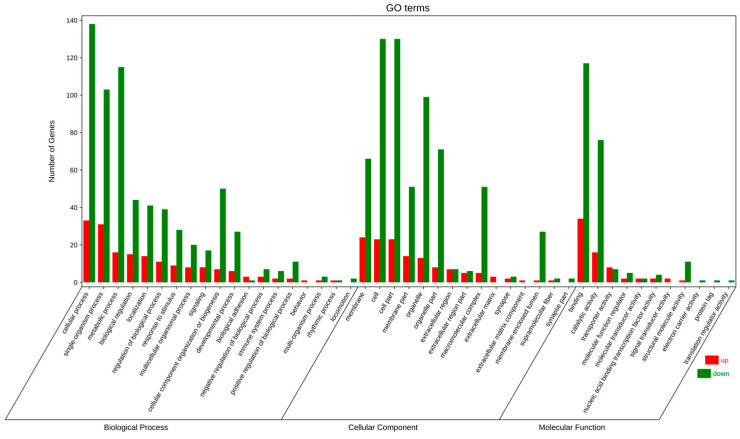
GO enrichment analysis of DEGs in HS vs. HT. The abscissa is the secondary GOterm, and the ordinate is the number of differential genes in the term. Red indicates upregulation and green indicates downregulation.

The analysis of a GO enrichment involves biological processes (BPs), cellular components (CCs), and molecular functions (MFs). The majority of functional annotation in the three categories showed that cellular processes, single-organism processes, and metabolic processes were mainly enriched in BPs and CCs, including membranes and cells; and in MFs, there was binding and catalytic activity (Figure 2 and Table S5). Among these DEGs, the main genes of interest are *slc2a5*, *prkaa2*, *pgp aacs*, and *odc1*, which are associated with glycan and lipid metabolism, antioxidant pathways, and cellular autophagy, respectively (Figure 1).

**Figure 3:**
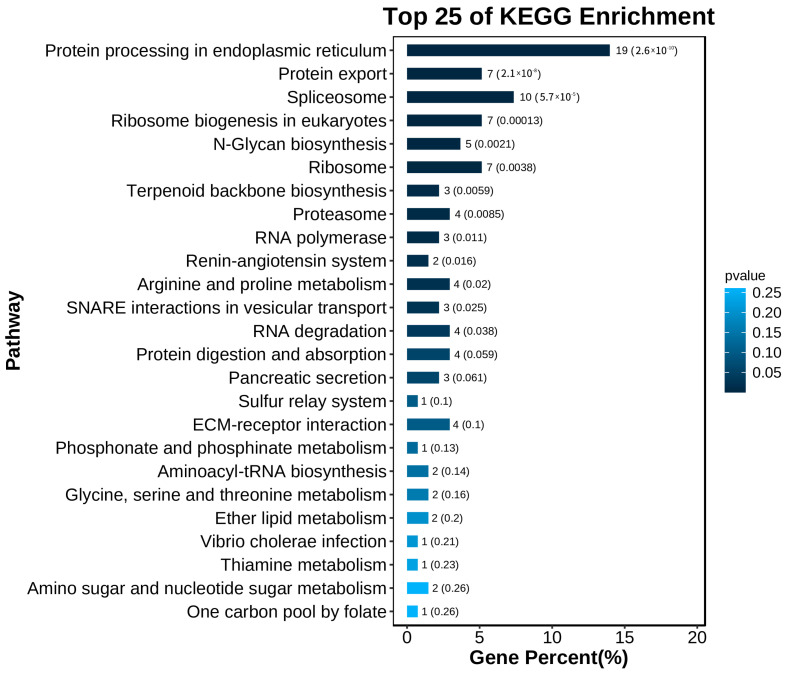
KEGG pathway annotation analysis of the top 25 pathways in HS vs. HT.

By KEGG analysis, DEGs from both groups were significantly enriched for six signaling pathways (Figure 3 and Table S6), including metabolism, genetic information processing, environmental information processing, cellular processes, organic systems, and human diseases. In metabolism, the main pathways are lipid metabolism, amino acid metabolism, and carbohydrate metabolism. Genetic information processing mainly includes translation and transcription processes. Environmental information processing mainly involves pathways related to signaling molecules, interactions, and signal transduction. Cellular processes are mainly related to transport and catabolism pathways and cell growth and death. In organic systems, the main pathways involved are the immune system, nervous system, and endocrine system, and human diseases include immune disease and cancer.

### 3.4. Validation of RNA-seq Results by qRT-PCR

**Figure 4:**
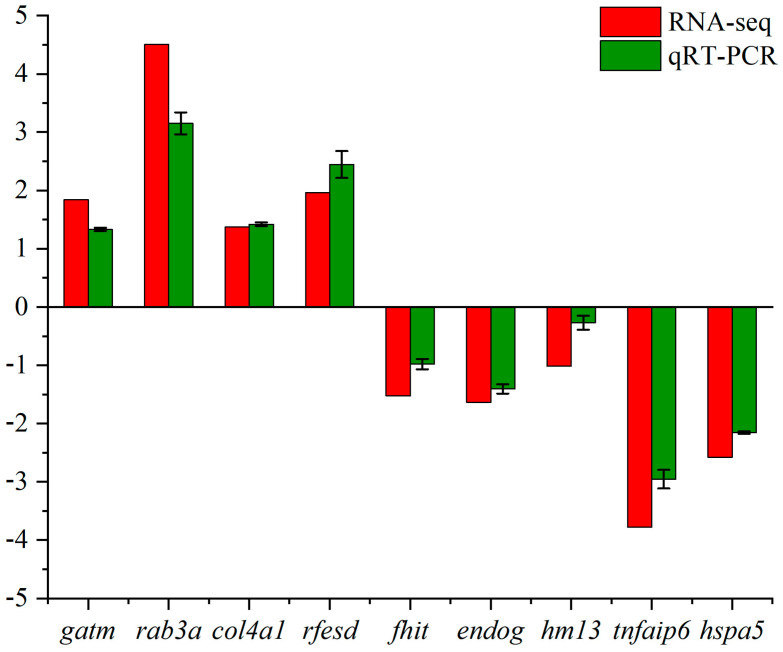
Comparison of expression levels for the 9 significantly expressed mRNAs using RNA-Seq and qRT-PCR. The X-axis represents the gene name, and the Y-axis represents the log2 value of the relative gene expression between the two groups. The length of the green column represents the average log2 value of the relative gene expression between the two groups by qPCR, and the length of the red column represents the average log2 value of the relative gene expression between the two groups by RNA-seq.

Nine genes were used to validate the results of RNA-seq. The results showed that the gene expression patterns obtained by both methods were consistent, which indicated that the results of transcriptome expression analysis were accurate and credible (Figure 4).

## 4. Discussion

Under the intolerance of greater amberjack to hypoxia, 829 DEGs associated with differential tolerance to acute hypoxia were screened by transcriptome sequencing, and enrichment analysis revealed the related key genes and biological pathways.

### 4.1. Glucose and Lipid Metabolism

In previous studies, it was found that in response to acute hypoxia, fish increase glucose uptake and utilization, as well as inhibit metabolic pathways that require oxygen and ATP [29]. In this study, it was found that *slc2a5* (solute carrier family 2 member 5), which encodes GLUT5, was upregulated in the HT group compared with the HS group. GLUT5 is a pro-transport protein with a high affinity for fructose and a low affinity for glucose, and it is mainly expressed in the liver and intestine [30]. Fructose is a monosaccharide that is mainly metabolized in the liver, where it can be further converted to glucose and participate in glycolysis and gluconeogenesis [31]. Under hypoxic conditions, aquatic animals undergo multiple metabolic pathways to adapt to the reduced oxygen environment. Switching from aerobic glycolytic metabolism to the glycolytic pathway to reduce oxygen consumption is a strategy to cope with a hypoxic environment and maintain energy supply [32]. As a result, there is an elevated demand for glucose, which, in turn, increases the amount of glucose transporter proteins in the cell membrane [33]. It has been reported that when faced with acute hypoxia, pearl gentian grouper and largemouth bass (*Micropterus salmoides*) will upregulate GLUT5 homologous family pro-transport proteins GLUT3 [21,34] and GLUT1 [33,35], respectively, to facilitate cellular glucose transport for energy. The difference in pro-glucose transporter protein species between greater amberjack and these two fish may be one of the reasons why it is so intolerant to hypoxia. *Slc2a5* was enriched as a single-organism process in GO analysis and was found to be associated with carbohydrate digestion and absorption in KEGG. In addition, the present study revealed a relative downregulation of *pgp* (phosphoglycerate phosphodiesterase) expression in the HT group, which has an inhibitory effect on gluconeogenesis. In primary rat hepatocytes, inhibition of G3PP (glycerol-3-phosphate phosphatase), encoded by *pgp*, caused a significant increase in the level of gluconeogenesis, while overexpression decreased the intensity of gluconeogenesis [36]. When fish are exposed to a hypoxic environment, the gluconeogenic pathway is enhanced to meet the energy requirements needed for metabolism. For example, in both euryoxic goby (*Gillichthys mirabilis*) [37] and Nile tilapia (*Oreochromis niloticus*) [38], enhanced gluconeogenic pathways were found, accompanied by a downregulation of *pgp* expression. Similarly, greater amberjack also downregulated *pgp* expression in the HT group, which may suggest that greater amberjack also maintains energy balance by enhancing the gluconeogenesis pathway to ensure proper energy supply for metabolism and replenish glucose or glycogen overconsumed by glycolysis under hypoxic stress. The *pgp* was enriched in the GO enrichment for metabolic processes, while in KEGG, it was found to be associated with carbon metabolism.

Lipids are an important source of energy and a major component of cell membranes in living organisms. Under prolonged hypoxic conditions, fatty acid synthesis pathways are inhibited in fish cells, while β-oxidation of fatty acids is enhanced to provide ATP to meet energy requirements [7,39]. In the present study, individuals in the HT group had upregulation of the *prkaa2* (AMP-activated protein kinase α2 subunit) gene, which has an inhibitory effect on lipid synthesis, relative to the HS group. AMPK (AMP-activated protein kinase) is a phylogenetically conserved intracellular metabolic regulator associated with glucose uptake and lipid metabolism [40]. Once activated, AMPK phosphorylates its downstream substrates to reduce ATP-consuming pathways, including fatty acid, cholesterol, and triacylglycerol synthesis, and increase ATP-producing pathways, such as fatty acid oxidation and glycolysis [41]. *Prkaa2* in the GO analysis was found to be associated with response to stimulus, while the KEGG analysis was enriched for the adipocytokine signaling pathway. In addition, a relative downregulation of *aacs* (acetoacetyl-CoA synthetase), which promotes lipid synthesis, was found in HT group individuals. *Aacs* is an acetoacetate-specific ligase that is involved in the generation of acetylaceto-coenzyme A for lipid biosynthesis in the cytoplasm [42]. In GO enrichment, *aacs* was found to be enriched for catalytic activity, and for KEGG analysis, they were shown to be associated with metabolic pathways. No genetic changes associated with increased lipid catabolism were found, which may be related to the acute hypoxia faced by greater amberjack and their low tolerance to hypoxia.

### 4.2. Antioxidant Effect and Apoptosis

The intracellular antioxidant system is activated in response to hypoxic stress for defense [43]. However, when this oxidative stress damage exceeds the physiological tolerance limit, it may lead to programmed cell death [44]. The antioxidant system in fish includes enzymatic antioxidants (glutathione peroxidase, GSH-Px; superoxide dismutase, SOD; catalase, etc.) and non-enzymatic antioxidants (glutathione, GSH; vitamins C and E, etc.). Among them, glutathione, an important antioxidant for plant and animal cells, can scavenge ROS, including hydroxyl radicals (OH) and singlet oxygen (^1^O_2_) [33].

In this study, the HT group of greater amberjack was found to produce a significant downregulation of the *odc1* gene relative to the HS group when exposed to hypoxic stress, resulting in the inhibition of antioxidant activity. ODC1 (ornithine decarboxylase1), encoded by *odc1*, is the rate-limiting enzyme for the synthesis of polyamines [45], which play a key role in cell proliferation, differentiation, and antioxidation [46]. Furthermore, the expression of *odc1* was significantly correlated with the antioxidant activity of GSH (glutathione). One study found that the GSH content in gray mullet (*Mugil cephalus*) tissues was significantly reduced in a hypoxic environment, suggesting that hypoxia induces the generation of oxidative stress. Moreover, the increase in the GSH content after reoxygenation implied that the antioxidant mechanism was activated [47]. In the pearl gentian grouper, when the *odc1* gene was significantly downregulated, the antioxidant activity of GSH was inhibited and the scavenging ability of ROS was reduced [21]. This suggests that acute hypoxia also induces oxidative stress in greater amberjack and may lead to a decrease in GSH antioxidant activity. In addition, the results of GO analysis showed that *odc1* was enriched in multicellular organismal processes, and the results of KEGG indicated that this gene is associated with glutathione metabolism.

It was found that *tnfaip3* and *irak3* were simultaneously downregulated in the HT group relative to the HS group after acute hypoxia. This may imply that individuals in the HT group have somewhat enhanced anti-apoptotic effects of the NF-kB pathway, thereby increasing tolerance to acute hypoxia. The coping mechanisms of hypoxic stress are closely related to NF-kappa B (NF-kB) signaling-mediated immune responses [39]. The NF-κB signaling pathway has an anti-apoptotic function and tends to be promoted when hypoxic stress is present [48]. The promotion of the NF-KB pathway has been found in gilthead seabream (*Sparus aurata*) [49], Manila clams (*Ruditapes philippinarum*) [50], and small abalone (*Haliotis diversicolor*) [51]. *Tnfaip3* (tumor necrosis factor alpha–induced protein 3) is a negative regulator of the NF-kB signaling pathway [52]. In blunt snout bream (*Megalobrama amblycephala*), the study also found that *tnfaip3* might contribute to hypoxia resistance [53]. *Irak3* (IRAK-M, interleukin-1 receptor-associated kinase 3) can similarly regulate NF-kB signaling through the Toll-like receptor signaling pathway [54]. In this study, two negative regulators of the NF-kB pathway were found to be simultaneously downregulated in the HT group relative to the HS group, which may be due to greater amberjack promoting the NF-kB pathway against apoptosis by repressing the expression of these two genes in response to oxidative stress. In GO and KEGG results, tnfaip3 was found to be enriched for the regulation of biological processes and the necroptosis pathway, while irak3 was associated with immune system processes and neurotrophin signaling pathways.

Furthermore, in the HT group, *endog*, *hm13*, *casp6*, *ptrh2*, *ptrh2*, and *fhit*, which activate autophagy and induce apoptosis [21,55,56,57,58], were also found to be downregulated relative to the HS group. This could mean that, after hypoxic stress is generated, greater amberjack would first “rescue itself” in these ways to mitigate the negative effects. In future experiments, we will further investigate the use of gills and other tissues to study and enhance our understanding of the molecular mechanism of hypoxia tolerance in greater amberjack.

## 5. Conclusions

Based on a comparative analysis of the liver transcriptome of acute hypoxia-tolerant and -intolerant groups in greater amberjack, we found that glycolipid metabolism, antioxidant activity, and apoptotic pathways significantly responded when exposed to acute hypoxia. Under acute hypoxia, greater amberjack may maintain normal physiological metabolism by promoting anaerobic glycolysis and inhibiting fatty acid synthesis to provide energy. Although antioxidant activity was inhibited and the apoptotic pathway was activated, the occurrence of anti-apoptotic effects through the promotion of the NF-kB pathway somewhat reduced the cellular damage caused by greater amberjack’s oxidative stress and prevented excessive apoptosis that affects tissue or organ function. Compared with the acute hypoxia-tolerant group, genes such as *slc2a5* and *prkaa2* related to promoting sugar transport and inhibiting lipid syntheses were upregulated, while genes that inhibit gluconeogenesis and promote lipid syntheses, such as *pgp* and *aacs*, were downregulated. The expression of *odc1* was significantly and relatively downregulated in the acute hypoxia-intolerant group, which would lead to the inhibition of intracellular antioxidant activity and decreased scavenging of ROS. The results of the present study elucidated the differences in liver transcriptome gene expression between the acute hypoxia-tolerant and acute hypoxia-intolerant groups of greater amberjack and revealed the molecular mechanisms underlying the response of greater amberjack to hypoxia. The hypoxia-related genes and pathways screened in this study provide potential targets for the future molecular breeding of hypoxia-tolerant greater amberjack varieties.

## Figures and Tables

**Table 1:** Summary of illumina RNA-seq data.

Sample	Raw_Reads	Clean_Reads	Clean_Bases	Error_Rate	Q20	Q30	GC_Pct (%)
1	47,274,858	44,974,632	6.75 G	0.03	96.49	90.94	48.85
2	46,793,042	43,993,002	6.6 G	0.03	96.53	90.92	49.15
3	45,919,032	43,032,780	6.45 G	0.03	96.9	91.75	49.65
4	47,007,730	44,086,276	6.61 G	0.03	96.37	90.58	48.31
5	43,530,104	41,775,200	6.27 G	0.03	96.81	91.54	48.85
6	46,781,622	43,964,854	6.59 G	0.03	96.61	91.16	49.68
7	47,396,944	44,495,152	6.67 G	0.03	96.79	91.46	49.65
8	45,488,812	42,321,398	6.35 G	0.03	96.85	91.6	49.67
9	50,102,200	46,354,446	6.95 G	0.03	96.95	91.87	49.44
10	45,492,008	43,214,822	6.48 G	0.03	96.64	91.22	49.56
11	51,245,632	46,862,522	7.03 G	0.03	96.93	91.8	49.66
12	47,303,766	44,999,926	6.75 G	0.03	96.67	91.23	49.47

## Data Availability

Data is contained within the article or supplementary material. The data presented in this study are available in [Tables S1–S6].

## Supplementary Materials

The following supporting information can be downloaded at: https://www.mdpi.com/article/10.3390/ani13172717/s1, Table S1. Sample information of greater amberjack. Table S2. Quantitative real-time PCR (qRT-PCR) primer sequences data. Table S3. All predicted genes of HT and HS. Table S4. Differentially expressed genes between HT and HS (|log2 (fold change) > 1.0| and *p*-value < 0.05). Table S5. Gene ontology (GO) of differentially expressed genes (DEGs) between HT and HS. Table S6. KEGG of differentially expressed genes (DEGs) between HT and HS.
